# Effects of Controlled-Release Fertiliser on Nitrogen Use Efficiency in Summer Maize

**DOI:** 10.1371/journal.pone.0070569

**Published:** 2013-08-02

**Authors:** Bin Zhao, Shuting Dong, Jiwang Zhang, Peng Liu

**Affiliations:** 1 State Key Laboratory of Crop Biology, College of Agronomy, Shandong Agricultural University, Tai’an, China; United States Department of Agriculture, United States of America

## Abstract

Nitrogen (N) is a nutrient element necessary for plant growth and development. However, excessive inputs of N will lead to inefficient use and large N losses to the environment, which can adversely affect air and water quality, biodiversity and human health. To examine the effects of controlled-release fertilisers (CRF) on yield, we measured ammonia volatilisation, N use efficiency (NUE) and photosynthetic rate after anthesis in summer maize hybrid cultivar Zhengdan958. Maize was grown using common compound fertiliser (CCF), the same amount of resin-coated controlled release fertiliser (CRFIII), the same amount of sulphur-coated controlled release fertiliser (SCFIII) as CCF, 75% CRF (CRFII) and SCF (SCFII), 50% CRF (CRFI) and SCF (SCFI), and no fertiliser. We found that treatments CRFIII, SCFIII, CRFII and SCFII produced grain yields that were 13.15%, 14.15%, 9.69% and 10.04% higher than CCF. There were no significant differences in grain yield among CRFI, SCFI and CCF. We also found that the ammonia volatilisation rates of CRF were significantly lower than those of CCF. The CRF treatments reduced the emission of ammonia by 51.34% to 91.34% compared to CCF. In addition, after treatment with CRF, maize exhibited a higher net photosynthetic rate than CCF after anthesis. Agronomic NUE and apparent N recovery were higher in the CRF treatment than in the CCF treatment. The N uptake and physiological NUE of the four yield-enhanced CRF treatments were higher than those of CCF. These results suggest that the increase in NUE in the CRF treatments was generally attributable to the higher photosynthetic rate and lower ammonia volatilisation compared to CCF-treated maize.

## Introduction

Nitrogen (N) is a critical element for plant growth, and adding N to crops is a valuable agronomic practice. During the past decade, China has made considerable progress in terms of grain yield (GY) and feeding its growing population; however, this increase in agricultural yield has partly resulted from excessive application of N fertilisers [1]. Excessive application can result in inefficiencies and large losses of excess N to the environment, which can impact air and water quality, biodiversity and human health [2]. The overuse of fertilisers contributes to NO_3_-N contamination of both surface water and soil water, and high profile NO_3_-N accumulation can reduce N use efficiency (NUE) [1], [3]. Releases of nitrous oxide (mainly via the application of N fertiliser) can degrade stratospheric ozone and contribute to global warming [4]. Ammonia (NH_3_) volatilisation from soil and plants can also aggravate environmental contamination and contribute to acid deposition [5]. Therefore, interventions to increase NUE and reduce N inputs are important not only for reducing environmental risk but also for lowering agricultural production costs [6].

Controlled-release fertiliser (CRF) is a possible alternative to common compound fertiliser (CCF) to increase N uptake efficiency and minimise N losses to the environment. However, current grower acceptance is limited due to a lack of experience with CRF performance and its high relative cost [7]. As one kind of enhanced-efficiency fertiliser, CRF has several advantages compared to CCF. Some of the advantages and disadvantages are listed in Table 1. The greatest benefits of switching from CCF to CRF include increased profitability and reductions in the environmental impact of crop production.

**Table 1:** Advantages and disadvantages of CRF over CCF.

Advantages	Disadvantages
Slower release rate – plants are able to take up most of the fertilisers	Very high costs
Reduced fertiliser loss – slower leaching and run-off	
Reduced labour capital – less frequent application is required	Lower consumption
Lower salt index – reduced plant damage from high concentrations of salts	
Fertiliser burn is not a problem with CRF even at high rates of application	Limited to nursery stock

Note(s): CRF, controlled-release fertiliser; CCF, common compound fertiliser.

In sandy nursery soils, CRF was shown to be effective for seedling production, due to the increased residence time of CRF in the soil relative to conventional fertilisation [8], [9]. Oliet et al. [10] found that CRF promoted suitable morphological values and nutritional status in *Pinus halepensis* planting stock, suggesting that the CRF types used in their study were suitable for the nursery production of *P*. *halepe*nsis. Tang et al. [11] reported major increases in rice yield following a single basal application of CRF, that was attributed to increased soil availability of N, superior development of the root systems, better nutrient absorption capacity, delayed senescence and enhanced lodging resistance.

To improve CRF, studies need to describe nutrient release characteristics. Du et al. [12] revealed that the thickness of the coating membrane was the most important parameter for controlling nitrate release, followed by temperature, granule radius and the saturated concentration of nitrate. However, the global use of CRF has so far been limited due to the higher cost (at least 2 or more times the price) compared to CCF.

To date, few investigations have been carried out in the field on the performance of crops grown with CRF. Even if CRF use becomes economical, the widespread acceptance by growers will likely be limited as a result of grower concern about field performance [7]. It has mainly been applied to nursery stock in foreign countries. Until now, reports on CRF in crops have focused mainly on domestic rice and little information is available about the effects of CRF on maize. Consequently, it is valuable to clarify the mechanisms of the impacts of CRF on maize. Therefore, in the present study, we investigated the following: how to select the right application rate of CRF in maize, the photosynthetic traits and physiological mechanisms associated with yields and the NUE of CRF in maize. Our results will assist in the successful application of CRF to maize fields.

## Materials and Methods

### Experimental Design

Summer maize hybrid Zhengdan958 (released in 2000) was planted on a farm at Shandong Agricultural University, Shandong Province, China (36°10′19′′N, 117°9′03′′E). The soil, classified as a silt loam, is considered to be highly suitable for crop production. The soil pH was 6.1. The average organic matter content in the tillage layer was 19.7 g kg^−1^ and the available N, phosphorus (P) and potassium (K) were 124.38 mg kg^−1^, 45.23 mg kg^−1^ and 81.78 mg kg^−1^, respectively. Methods of soil analysis referenced from “Agricultural Soil Analysis” written by Bao S D [13]. Two kinds of CRF, a resin-coated CRF (hereafter, CRF) and a sulphur-coated CRF (hereafter, SCF) offered by Shandong Kingenta Ecological Engineering Co., Ltd. located in No. 19 Xingda West Street, Linshu, Shandong Province were used in our experiment and common compound fertiliser (CCF) was used as a control. The content of N, P_2_O_5_ and K_2_O in each CCF, CRF and SCF was 24%, 8% and 16%; 21%, 7% and 14%; and 18%, 6% and 12%, respectively. The experiment was conducted as a random complete block design with three replications. There were eight treatments: CCF applied at 1250 kg ha^−1^ (the local average commercial fertiliser N application rate; hereafter, CCF); CRF applied at 714.29 kg ha^−1^ (CRFI, 50% CCF), 1071.43 kg ha^−1^ (CRFII, 75% CCF) and 1428.57 kg ha^−1^ (CRFIII, 100% CCF); SCF applied at 833.33 kg ha^−1^ (SCFI, 50% CCF), 1250 kg ha^−1^ (SCFII, 75% CCF) and 1666.67 kg ha^−1^ (SCFIII, 100% CCF); and control plots without fertiliser application (CK). All fertilisers were applied at a basal dose.

### Measurement of Net Photosynthetic Rate

Net photosynthetic rate (*P*_N_) was measured with a portable, open-flow portable photosynthetic system (LI-COR, LI-6400 System, UK) in 2006. The photosynthetic photon flux density (PPFD) was 1400 µmolm^−2^s^−1^ provided by the internal light source of the leaf chamber. The measurements were taken at approximately 10-day intervals following pollination on cloudless days. All of the leaves (22 or 23) were fully expanded at the time of measurement. Only ear leaves were used for *P*_N_ measurements, because these structures are metabolically active for the longest period of time and their relative contribution to total photosynthetic assimilates is high [14]. The leaves for experiments were all fully exposed and oriented to normal irradiation during measurements. Five plants per treatment were randomly selected for measurements.

### Plant Sampling and N Content Determination

To measure aboveground N uptake, five plants were collected per treatment in about 10-day intervals 30 days after sowing. At the mature stage, five plants were manually harvested per treatment. Rows per ear (RE), kernels per row (KR) and kernels per ear (KE) were counted. Aboveground dry matter (DM) was determined by oven-drying the samples at 80°C until a constant weight was achieved. Subsequently, samples were manually separated into the vegetative and grain portions. Then the GY and thousand-kernel weight (TKW) were determined.

The grains and straw were ground using a cyclone sample mill with a mesh size of 0.5 mm. Then the grain N concentration (GNCT) and the straw N concentration (SNCT) were measured using the micro-Kjeldahl method (CN61M/KDY-9820, Beijing).

The following parameters were calculated:
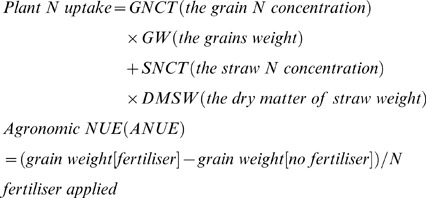


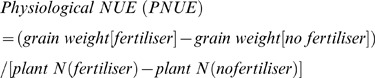


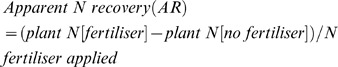


The plant N included N in dry matter of straw and grain.

### Ammonia (NH_3_) Volatilisation Measuring Device and Procedure

#### Devices

**Figure 1:**
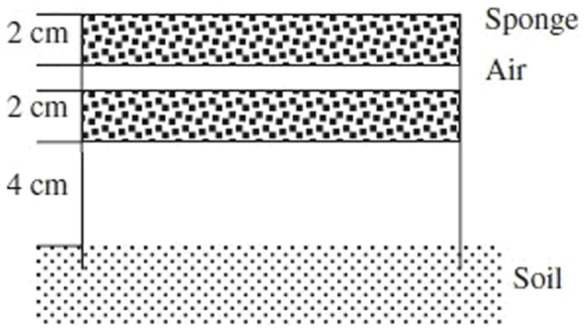
Vented-chamber methods used in field experiments to capture NH_3_ emitted from the soil.

The vented chamber (Figure 1) was made of grey round polyvinyl chloride (PVC) tubing (15 cm internal diameter and 10 cm high), as described by Liao [15]. Two pieces of round sponge (16 cm in diameter and 2 cm in thickness) were moistened with a 15 mL phosphate-glycerol solution (50 mL analytical phosphate and 40 mL glycerol diluted to 1000 mL with pure water) and then inserted into each chamber. Because the volume of the solution only accounted for 3.7% of the sponge’s volume, the sponge was still ventilative after being moistened. The sponge inside the chamber absorbed NH_3_ volatilised from the soil, and the top sponge absorbed NH_3_ from the ambient air. Glycerol in the sponges absorbed moisture from the air inside or outside the chamber and prevented the sponges from drying [16].

### Detection of Ammonia Volatilization in Field

On June 12, 2006, when N fertiliser was applied to summer maize at a basal dose, five sets of both vented chambers were evenly placed at different locations in each plot, with the bottom edge pushed 2 cm into the soil. The two pieces of sponge in the vented chamber and the boric acid solution in the closed chamber were replaced every day during the first week and every 2 to 3 days during the second and third week. Simultaneously, each chamber was moved to a new location in the plot. Ammonia in the phosphate solution in each sponge inside the vented chamber was extracted with 300 mL of 1 M KCl after 60 min of oscillation. Ammonium quantities in the KCl extract solution were measured using the micro-Kjeldahl method (CN61M/KDY-9820, Beijing). NH_3_ volatilisation from the soil was estimated by the following formula:
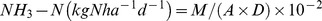
where M is the NH_3_ (mg N) captured by the vented chamber during each sampling, A is the cross-section area (m^2^) of the round chamber, D (days) is the duration of each sampling, and 10^−2^ is 10,000 m^2^ ha^−1^×10^−6 ^kg mg^−1^.

In the field, five sets of vented-chamber devices were evenly placed at different locations in each plot in the manner described above. During the summer maize-growing season, NH_3_ emission was measured from June 12 to August 3, 2006, after basal N fertilisation. Measurements continued for 53 days.

### Statistical Analysis

Statistical analyses were performed using the analysis of variance (ANOVA) in the General Linear Model procedure of SPSS (Ver. 11, SPSS, Chicago, IL, USA). Results are presented as means of the 2 years of experimentation, because the trends of these parameters were consistent between years. The least significant differences (LSDs) between the means were estimated at the 95% confidence level. Unless indicated otherwise, significant differences among different plants are given at *P*<0.05. LSD was used to compare adjacent means arranged in order of magnitude. Calculations and linear regressions were performed using a *SigmaPlot 10*.*0* program.

## Results

### GY and GY Components

The application of fertilisers increased GY significantly compared to that of no fertiliser (Table 2), and the effect of CRF was much more pronounced than that of CCF. Furthermore, CRFIII, SCFIII, CRFII and SCFII were 13.15%, 14.15%, 9.69% and 10.04% higher in GY than CCF. No significant difference in GY was found between CRFI, SCFI and CCF, and there was no significant difference in GY between the two CRFs. The average economic efficiency of CRFIII/SCFIII was 1190.50 yuan hm^−2^ more than CCF; CRFII/SCFII was 1753.75 yuan hm^−2^ more than CCF; CRFI/SCFI was 758.75 yuan hm^−2^ more than CCF.

**Table 2:** Effect of controlled-release fertiliser on yield and its component of summer maize.

Treatments	Rows per ear	Kernels per row	Kernels per ear	Wt. per 1000-kernel(g)	Grain yield (kg hm^−2^)		Benefit increase than CK (yuan hm^−2^)	
					2005	2006	2005	2006
CK	14.44	39.02	563.65	281.17e	9383.8d D	8896.1c D	–	–
CCF	14.95	40.74	609.02	293.72d	11380.4c C	11046.1b BC	1056e	1349e
CRFI	14.53	39.93	580.36	298.20cd	11558.6bc BC	10826.8b C	2257c	1790d
CRFII	15.20	40.83	620.67	300.71c	12382.7ab AB	11990.4a A	2882a	3064a
CRFIII	14.60	41.70	608.82	316.29a	12716.9a A	12108.0a A	2571b	2339b
SCFI	14.67	40.57	595.04	296.58cd	11331.8c C	10986.4b BC	1763d	2035c
SCFII	14.80	42.07	622.59	299.77cd	12523.5a AB	11909.5a AB	3060a	2819a
SCFIII	15.07	41.16	620.08	308.53b	12810.5a A	12011.0a A	2629b	2033c

CCF, common compound fertiliser; CRF, a resin-coated CRF; SCF, a sulphur-coated CRF.

CCF, applied at 1250 kg ha^−1^ (the local average commercial fertiliser N application rate); CRFI, CRF applied at 714.29 kg ha^−1^ (50% CCF), CRFII,1071.43 kg ha^−1^ (75% CCF), CRFIII, 1428.57 kg ha^−1^ (100% CCF); SCFI, SCF applied at 833.33 kg ha^−1^ (50% CCF), SCFII, 1250 kg ha^−1^ (75% CCF), SCFIII, 1666.67 kg ha^−1^ (100% CCF); CK, control plots without N application.

Yield component of summer maize includes rows per ear, kernels per row, kernel No. per ear and Wt. per 1000-kernel.

According to the average market price at present, that is 1911 yuan t^−1^ for maize, 2190 yuan t^−1^ for CCF, 2660 yuan t^−1^ for CRF, 2350 yuan t^−1^ for SCF.

All data are means of 3 replications.

Means values marked with different capital letters indicate significant differences at *P* = 0.01 level; different small letters indicate significant differences at *P* = 0.05 level.

### Net Photosynthetic Rate (Post-anthesis Changes in the Light-saturated Photosynthesis Rate)

**Figure 2:**
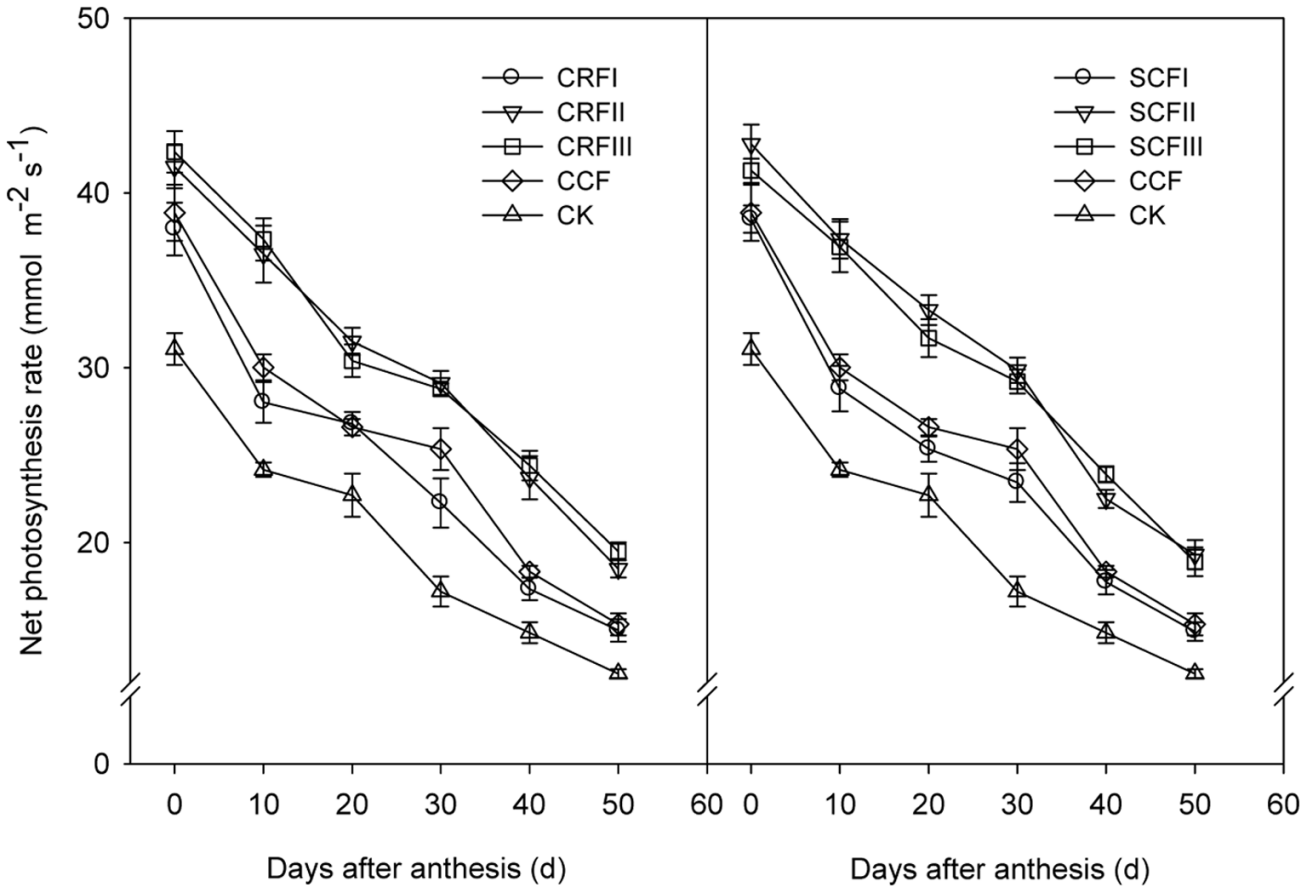
Effects of controlled-release fertiliser on net photosynthetic rate in ear leaves of summer maize. CCF, common compound fertiliser; CRF, a resin-coated CRF; SCF, a sulphur-coated CRF; CCF, applied at 1250 kg ha^−1^ (the local average commercial fertiliser N application rate); CRFI, CRF applied at 714.29 kg ha^−1^ (50% CCF), CRFII,1071.43 kg ha^−1^ (75% CCF), CRFIII, 1428.57 kg ha^−1^ (100% CCF); SCFI, SCF applied at 833.33 kg ha^−1^ (50% CCF), SCFII, 1250 kg ha^−1^ (75% CCF), SCFIII, 1666.67 kg ha^−1^ (100% CCF); CK, control plots without N application. Error bars are SE (n = 5).

There was no significant difference in net *P*_N_ among treatments (Figure 2). All *P*_N_ of the ear leaves decreased after flowering, and *P*_N_ values for CRFIII, CRFII, SCFIII and SCFII decreased more slowly than CCF. The *P*_Ns_ of CRFIII, CRFII, SCFIII and SCFII on the 10^th^ day after flowering were 24.4%, 21.6%, 23.0% and 24.5%, higher, respectively, than those of CCF (*P*<0.05); on the 30^th^ day after flowering were 13.6%, 14.9%, 15.3% and 17.8%, higher, respectively, than those of CCF (*P*<0.05); on the 50^th^ day after flowering were 27.3%, 20.8%, 23.4% and 26.0%, higher, respectively, than those of CCF (*P*<0.05). No significant difference in *P*_N_ was observed among CRFI, SCFI and CCF. These results suggest that the yield increases afforded by the CRF treatments were generally attributable to their higher photosynthetic rates.

### NH_3_ Volatilisation

**Figure 3:**
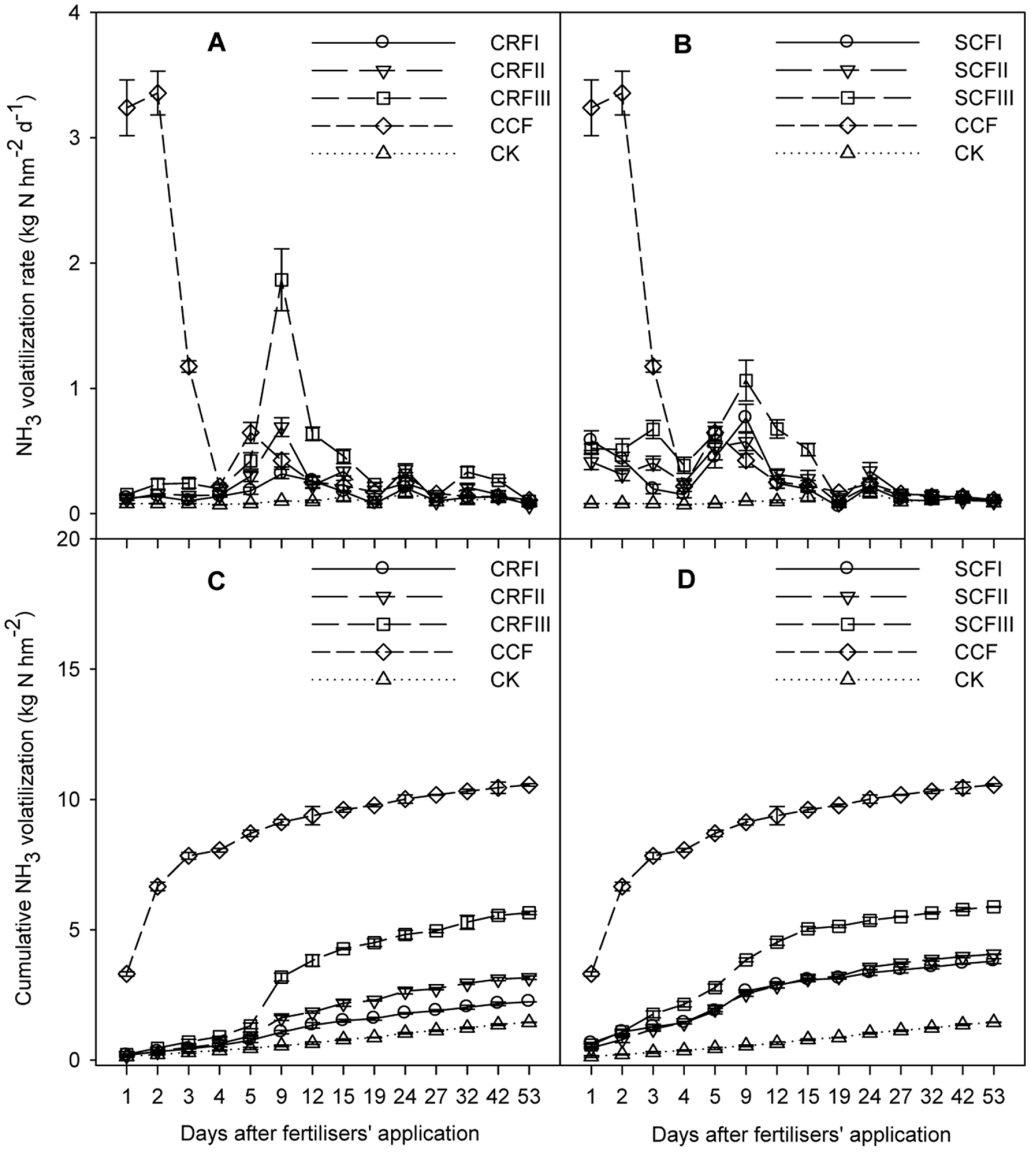
Soil NH_3_ volatilisation rates (A and B) and changes in cumulative NH_3_ volatilisation (C and D) following basal fertilisation. CCF, common compound fertiliser; CRF, a resin-coated CRF; SCF, a sulphur-coated CRF; CCF, applied at 1250 kg ha^−1^ (the local average commercial fertiliser N application rate); CRFI, CRF applied at 714.29 kg ha^−1^ (50% CCF), CRFII,1071.43 kg ha^−1^ (75% CCF), CRFIII, 1428.57 kg ha^−1^ (100% CCF); SCFI, SCF applied at 833.33 kg ha^−1^ (50% CCF), SCFII, 1250 kg ha^−1^ (75% CCF), SCFIII, 1666.67 kg ha^−1^ (100% CCF); CK, control plots without N application. Error bars are SE (n = 15; some SE bars are smaller than the symbols).

The application of N fertilisers in the field increased the volatilisation of NH_3_ (Figure 3). The maximum flux of NH_3_ increased to 3.36 kg N ha^−1^ d^−1^ 2 days after the application of CCF, and then rapidly decreased to approximately 1.18 kg N ha^−1^ d^−1^. However, the flux of NH_3_ from CRF treatments was significantly lower than that of the CCF treatment. NH_3_ volatilisation fluxes from CRF treatments peaked later than those of CCF. The treatments CRFIII and SCFIII reached their peak NH_3_ volatilisation fluxes of 1.87 kg N ha^−1^ d^−1^ and 1.06 kg N ha^−1^ d^−1^, respectively, 9 days following fertiliser application (Figure 3 A B).

Cumulative rates of NH_3_ volatilisation generally displayed similar patterns of increase and temporal characteristics up to 53 days following treatment (Figure 3 C D), after which parameters remained relatively constant. Cumulative fluxes of NH_3_ emitted from the field were 10.56 kg N ha^−1^ d^−1^, 2.23 kg N ha^−1^ d^−1^, 3.16 kg N ha^−1^ d^−1^, 5.65 kg N ha^−1^ d^−1^, 3.79 kg N ha^−1^ d^−1^, 4.06 kg N ha^−1^ d^−1^ and 5.88 kg N ha^−1^ d^−1^ in the CCF, CRFI, CRFII, CRFIII, SCFI, SCFII and SCFIII treatments, respectively. Volatilisation rates of NH_3_ were 78.8%, 70.0%, 46.5%, 64.1%, 61.5% and 44.3% lower than those of CCF for CRFI, CRFII, CRFIII, SCFI, SCFII and SCFIII, respectively (*P*<0.05). These results suggest that the application of CRF considerably decreased NH_3_ volatilisation rates.

### N Uptake and NUE

**Figure 4:**
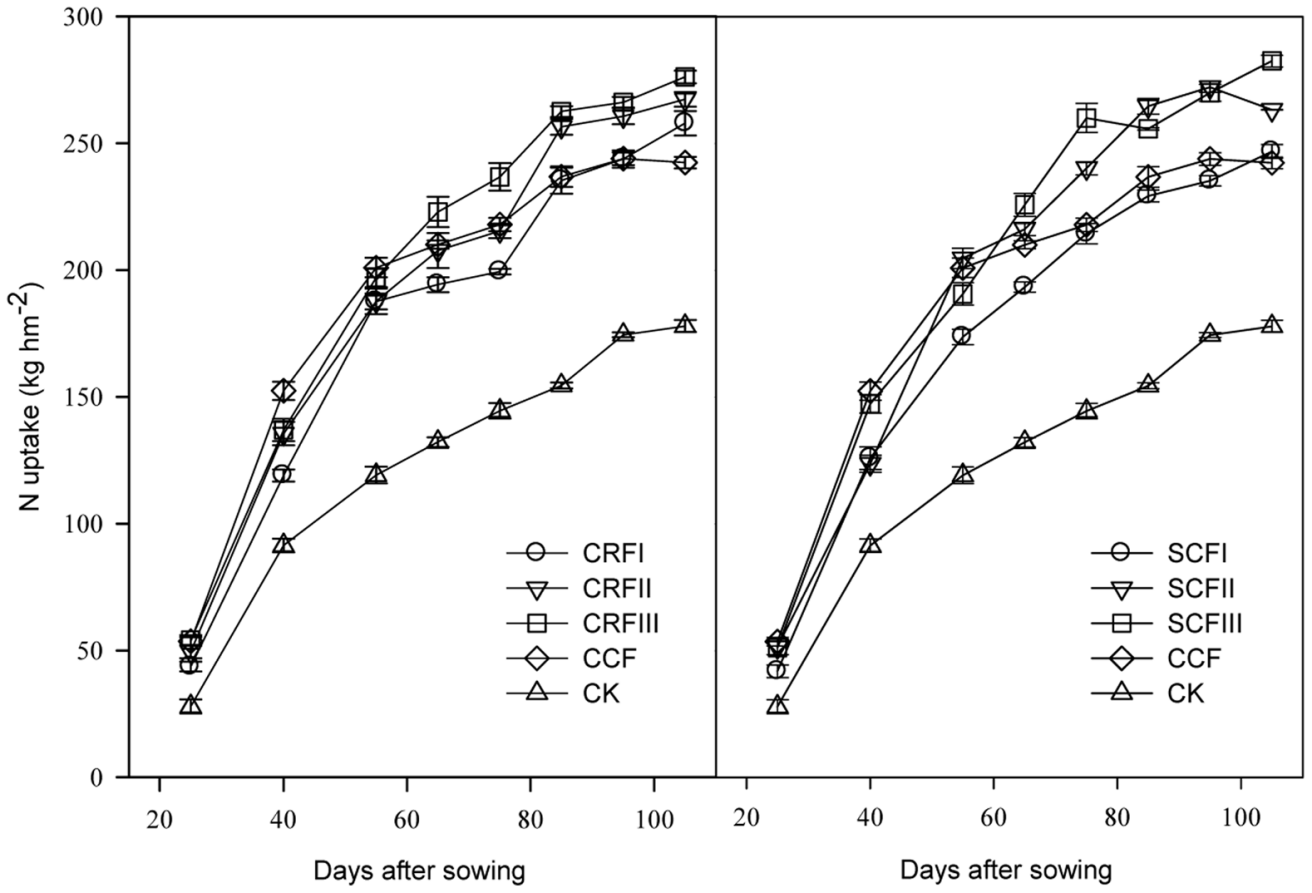
Dynamics of N uptake by aboveground parts after fertilisation. CCF, common compound fertiliser; CRF, a resin-coated CRF; SCF, a sulphur-coated CRF; CCF, applied at 1250 kg ha^−1^ (the local average commercial fertiliser N application rate); CRFI, CRF applied at 714.29 kg ha^−1^ (50% CCF), CRFII,1071.43 kg ha^−1^ (75% CCF), CRFIII, 1428.57 kg ha^−1^ (100% CCF); SCFI, SCF applied at 833.33 kg ha^−1^ (50% CCF), SCFII, 1250 kg ha^−1^ (75% CCF), SCFIII, 1666.67 kg ha^−1^ (100% CCF); CK, control plots without N application. Error bars are SE (n = 3; some SE bars are smaller than the symbols).

Following the field application of CCF, N uptake increased rapidly during the first phase (i.e., the stage prior to flowering) and then increased slowly after flowering (Figure 4). Phenotypic phenomena such as vigorous growth before flowering and premature senescence after flowering were observed (data not shown). However, N uptake increased relatively constantly with the increased application of CRF throughout the growth period (Figure 4), and caused obvious delays in leaf senescence. After flowering, the rank of shoot N uptake among all treatments was CRFIII>CRFII>CRFI>CCF>CK and SCFIII>SCFII>CCF>SCFI>CK.

ANUE and NR were significantly higher for CRF than for CCF (91.7% and 89.8%, respectively; *P*<0.05) (Table 3). Although no significant differences in PNUE were found between CRF and CCF, PNUE was slightly higher for CRFIII, CRFII, SCFIII and SCFII than for CCF.

**Table 3:** Effects of different controlled-release fertiliser treatments on NUE of maize.

Treatments	Grain yield (t hm^−2^)	Total N uptake (kg N hm^−2^)	Agronomic N use efficiency (kg_grain_ kg^−1^N)	Apparent N recovery (%)	Physiological N use efficiency (kg_grain_ kg^−1^N)
CK	9.38d	177.83	–	–	–
CCF	11.38c	242.3	6.66c	21.49f	30.97ab
CRFI	11.56bc	257.82	14.50a	53.33a	27.19b
CRFII	12.38ab	267.28	12.56ab	39.76c	31.59ab
CRFIII	12.72a	276.11	11.11b	32.76e	33.92ab
SCFI	11.33c	246.82	12.99ab	45.99b	28.24b
SCFII	12.52a	263.28	13.95ab	37.98cd	36.74a
SCFIII	12.81a	282.48	11.42ab	34.88de	32.74ab

CCF, common compound fertiliser; CRF, a resin-coated CRF; SCF, a sulphur-coated CRF; CCF, applied at 1250 kg ha^−1^ (the local average commercial fertiliser N application rate); CRFI, CRF applied at 714.29 kg ha^−1^ (50% CCF), CRFII,1071.43 kg ha^−1^ (75% CCF), CRFIII, 1428.57 kg ha^−1^ (100% CCF); SCFI, SCF applied at 833.33 kg ha^−1^ (50% CCF), SCFII, 1250 kg ha^−1^ (75% CCF), SCFIII, 1666.67 kg ha^−1^ (100% CCF); CK, control plots without N application.

All data are means of 3 replications.

Means values marked with different letters indicate significant differences at *P* = 0.05 level.

## Discussion

### Suitable Application Rates for CRF in the Field

Fertiliser use efficiency has become a critical measure of sustainable agriculture. Efforts are underway to improve crop production and enhance N use efficiency in two main ways: by breeding new varieties of maize with high NUE and by improving fertiliser application management [17], [18]. Among the improved N management practices, the use of enhanced-efficiency fertilisers such as SRF and CRF, nitrification inhibitors (NI) and urease inhibitors (UI) are being studied extensively under a variety of environmental conditions and agricultural systems to determine their effectiveness for increasing agricultural production and reducing environmental N losses [19]. CRFs are the fertilisers of the future, especially for open-field crops. Therefore, choosing the appropriate application rate is critical for the successful field application of CRF. In the present study, values of GY were not significantly different between CRFI/SCFI and CCF, CRFIII/SCFIII or CRFII/SCFII. However, values of GY for CRFIII, CRFII, SCFIII and SCFII were significantly higher than for CCF. Furthermore, agronomic NUE, apparent N recovery and economic efficiency of fertiliser for CRFII/SCFII (equivalent to 75% of CRF/SCF) were higher significantly than CCF. These results suggest that both of the two CRF types used in this study were effective for the agricultural production of maize, which also indicates that the CRFII/SCFII treatments corresponded to the optimum application rate of CRF for the maize fields studied in the North China Plain.

### Photosynthesis Rates and Physiological Mechanisms of NUE

Active photosynthesis has always been considered a desirable characteristic during the growing season [20]. Active photosynthesis in maize is primarily associated with the plants’ ability to produce grain [20], [21]. It had been suggested that yield increases in maize may be at least partly accounted for by increases in net leaf photosynthesis [22]. Leaf photosynthesis has been studied extensively as a plant trait in relation to NUE [23].

In the present study, after flowering, *P*_N_ in the yield-enhanced treatments (CRFIII, SCFIII, CRFII and SCFII) was significantly higher than that in the CCF treatments. No significant difference was found in *P*_N_ and yield among CRFI, SCFI and CCF. This suggests that the yield increase in the four CRF treatments may be attributable to the higher net photosynthetic rate. Wang et al. [14] also suggested that a yield increase in two cross-pollination treatments was generally due to a higher photosynthetic rate and related photosynthetic traits. Delaying or slowing down senescence may improve yield by increasing photosynthetic leaf area, which increases total photosynthate transported to sink tissue [24]. Indeed, in the present study, phenotypic delayed leaf senescence was obvious in the fourth yield-enhanced treatments (CRFIII, SCFIII, CRFII and SCFII), in accordance with Iain et al. [24].

### Reduced NH3 Volatilisation

Ammonium ions (NH_4_^+^) in the soil exist in equilibrium with NH_3_. If this conversion occurs at the soil surface and is accompanied by warm sunny days, NH_3_ is subject to gaseous losses to the atmosphere. NH_3_ is the most prolific atmospheric reactive N species emitted [25], and agricultural NH_3_ emissions are predicted to increase significantly in Asia from 13.8 Tg N year^−1^ in 2000 to 18.8 Tg N year^−1^ in 2030 [26]. NH_3_ emissions may result in N deposition to neighbouring ecosystems, which can damage vegetation [27]. In addition, some of the NH_3_ may be oxidised and converted into nitric acid, which together with sulphuric acid, make up acid rain. This acidic deposition also damages vegetation, and can acidify both soil and surface water, inducing aluminium toxicity in terrestrial and aquatic organisms [28]. NUE are GY are complex traits that depend on interactions among several component traits [18]. Both appear to be most affected by the NH_3_ volatilisation losses of N fertilisers [19].

NH_3_ volatilisation in the present study was highest for the CCF treatment, and the majority of N losses occurred within the first 2–12 days after CCF application. However, the majority of N losses occurred within the first 9–20 days after CRF application. The measured N uptake rates in the CRFIII, SCFIII, CRFII and SCFII treatments were higher than those of the CCF treatment, and ANUE and NR were significantly higher for CRF than for CCF. This suggests that ANUE and NR are significantly and positively correlated with N uptake, and negatively correlated with NH_3_ volatilisation. Improvements in NUE and environmental protection are increasingly important issues. The use of CRF can partially resolve both of these issues. The reduced NH_3_ volatilisation of CRF will help improve NUE while also decreasing the environmental contamination associated with excess N leaching. In addition, we found that the residual N of CRF in 0–100 cm soil profile was significantly higher than that of CCF (data not shown). Therefore, we conclude that CRF can be used to help conserve both air and water quality by maximising NUE and reducing N losses to the environment.

## Conclusion

We found that GY was significantly higher for CRFIII, CRFII, SCFIII and SCFII treatments than for CCF treatment, while no significant difference in GY was found between CRFIII/SCFIII or CRFII/SCFII. These results indicate that 75% CRF/SCF was the optimum application rate for CRF in maize fields of the North China Plain. Further research is necessary to determine the effects, if any, of the type, frequency and timing of CRF applications on maize. In addition, the yield increases afforded by CRF were partly due to higher rates of net photosynthesis and lower rates of NH_3_ volatilisation. Finally, we need to develop integrative approaches for enhancing societal *acceptance* of CRF, and for promoting the global application of this technology in economically sound and environmentally friendly agricultural systems.

## References

[1] Zhu ZL, Chen DL (2002) Nitrogen fertilizer use in China contributions to food production, impacts on the environment and best management strategies. Nutrient Cycling in Agroecosystems 63: 117–127.

[2] Goulding K, Jarvis S, Whitmore A (2008) Optimizing nutrient management for farm systems. Philosophical Transactions of the Royal Society B: Biological Sciences 363: 667–680. 10.1098/rstb.2007.2177 PMC2610177 17652069

[3] Ju X, Liu X, Zhang F, Roelcke M (2004) Nitrogen fertilization, soil nitrate accumulation, and policy recommendations in several agricultural regions of China. AMBIO: A Journal of the Human Environment 33: 300–305. 10.1579/0044-7447-33.6.300 15387063

[4] Milich L (1999) The role of methane in global warming: where might mitigation strategies be focused? Global Environmental Change, Part A: Human and Policy Dimensions 9: 179–201.

[5] Harper L, Sharpe R (1995) Nitrogen dynamics in irrigated corn: soil-plant nitrogen and atmospheric ammonia transport. Agronomy Journal 87: 669–675.

[6] Wang RF, An DG, Hu CS, Li LH, Zhang YM, et al (2011) Relationship between nitrogen uptake and use efficiency of winter wheat grown in the North China Plain. Crop & Pasture Science 62: 1–11.

[7] Medina CL, Obreza TA, Sartain JB, Rouse RE (2008) Nitrogen release patterns of a mixed controlled-release fertilizer and its components. Hort Technology 18: 475–480.

[8] Dobrahner J, Lowery B, Iyer JG (2007) Slow-release fertilization reduces nitrate leaching in bareroot production of Pinus strobes seedlings. Soil Science 172: 242–255.

[9] Zotarelli L, Scholberg JM, Dukes MD, Munoz-Carpena R (2008) Fertilizer residence time affects nitrogen uptake efficiency and growth of sweet corn. Journal of Environmental Quality 37: 1271–1278. 18453447 10.2134/jeq2007.0460

[10] Oliet J, Planelles R, Segura ML, Artero F, Jacobs DF (2004) Mineral nutrition and growth of containerized Pinus halepensis seedlings under controlled-release fertilization. Sci Hortic (Amsterdam) 103: 113–129.

[11] Tang SH, Yang SH, Chen JS, Xu PZ, Zhang FB, et al (2007) Studies on the mechanism of single basal application of controlled-release fertilizers for increasing yield of rice (Oryza sativa L.). Agricultural Sciences in China 6: 586–596.

[12] Du C, Tang D, Zhou J, Wang H, Shaviv A (2008) Prediction of nitrate release from polymer-coated fertilizers using an artificial neural network model. Biosyst. Eng. 99: 478–486.

[13] Bao SD (2005) Agricultural Soil Analysis [M]. Beijing:China Agriculture Press.

[14] Wang RF, An DG, Xie QE, Wang KJ, Jiang GM (2009) Leaf photosynthesis is enhanced in normal oil maize pollinated by high oil maize hybrids. Industrial Crops and Products 29: 182–188.

[15] Liao XL (1983) The methods of research of gaseous loss of nitrogen fertilizer. Progress Soil Sci. 11: 49–55.

[16] Wang CH, Liu XJ, Ju XT, Zhang FS, Malhi SS (2004) Ammonia Volatilization Loss from Surface-Broadcast Urea: Comparison of Vented- and Closed-Chamber Methods and Loss in Winter Wheat–Summer Maize Rotation in North China Plain. Communications in Soil Science and Plant Analysis 35: 2917–2939.

[17] Baligar V, Fageria N, He Z (2001) Nutrient use efficiency in plants. Communications in Soil Science and Plant Analysis 32: 921–950.

[18] Dawson J, Huggins D, Jones S (2008) Characterizing nitrogen use efficiency in natural and agricultural ecosystems to improve the performance of cereal crops in low-input and organic agricultural systems. Field Crops Research 107: 89–101.

[19] Motavalli PP, Goyne KW, Udawatta R (2008) Environmental impacts of enhanced efficiency nitrogen fertilizers. Online. Crop Management. doi:10.1094/CM-2008-0730-02-RV.

[20] Ding L, Wang KJ, Jiang GM, Liu MZ, Niu SL, et al (2005) Post-anthesis changes in photosynthetic traits of maize hybrids released in different years. Field Crop Research 93: 108–115.

[21] Araya T, Noguchi K, Terashima I (2006) Effects of carbohydrate accumulation on photosynthesis differ between sink and source leaves of Phaseolus vulgaris L. Plant & Cell Physiology. 47: 644–652. 10.1093/pcp/pcj033 16540483

[22] Dwyer LM, Tollenaar M (1989) Genetic improvement in photosynthetic response of hybrid maize cultivars 1959 to 1988. Can.J. Plant Sci. 69: 81–91.

[23] Foulkes M, Hawkesford M, Barraclough P, Holdsworth M, Kerr S, et al (2009) Identifying traits to improve the nitrogen economy of wheat: Recent advances and future prospects. Field Crops Research 114: 329–342.

[24] Iain SD, Alan PG, Howard T, Keith JE, David E, et al (2007) Modification of nitrogen remobilization, grain fill and leaf senescence in maize (Zea mays) by transposon insertional mutagenesis in a protease gene. New Phytology 173: 481–494. 10.1111/j.1469-8137.2006.01928.x 17244043

[25] Galloway JN, Cowling EB (2002) Reactive nitrogen and the world: 200 years of change. Ambio 31: 64–71. 12078011 10.1579/0044-7447-31.2.64

[26] Zheng XH, Fu CB, Xu XK, Yan XD, Huang Y, et al (2002) The Asian nitrogen cycle case study. Ambio 31: 79–87. 12078013 10.1579/0044-7447-31.2.79

[27] Newbould P (1989) The use of fertiliser in agriculture. Where do we go practically and ecologically? Plant Soil 115: 297–311.

[28] Reuss JO, Johnson DW(1986) Acid deposition and acidification of soil and waters. Ecol Studies No. 59. Springer-Verlag, NY.

